# Supplementary material for: Tracking diachronic sentiment change of economic terms in times of crisis: Connotative fluctuations of ‘inflation’ in the news discourse
Source: PLoS One. 2023 Nov 2;18(11):e0287688. doi: 10.1371/journal.pone.0287688 (PMC10621966; doi:10.1371/journal.pone.0287688)
Supplement: Attachment — Submitted filename: notes_review_inflation - Copy.docx [file pone.0287688.s002.docx]

Comments on the question: “Is the manuscript technically sound, and do the data support the conclusions?”

The issue here is that the data (corpus) is not adequately described by the authors. Much more information is necessary to describe the corpus design and composition (of the Great Recession News Corpus) and to evaluate its representativeness. The reader does not know which newspapers are included, how many texts from each newspaper there are per year, the readership and political orientation of the included newspapers, etc. No explanation is given of the file names and what they mean. In lines 303-304 the hypotheses are not spelt out more fully.

Regarding the statistical analysis and methods, while some aspects are fully described, others do rely on readers already being familiar with the relevant stats measure/method (for example, the ‘peaks-and-troughs’ and UFA; Gwet’s AC1 agreement statistic). It would be good if the authors could review what they have written and ensure that all aspects/methods are adequately explained to those who are unfamiliar with them. It would also be good if explicit critical method reflection was included, for example in the Conclusion (what can these methods not show us? What are their shortcomings?) It was also unclear to me whether individual variation (e.g. between newspaper articles, authors, newspapers, newspaper type) was included in the statistical analysis. No information on such issues is in the tables/results, which just appears to provide aggregate information per year. Is there any effect of the type of news item (eg opinion vs news), the voice who produces the word “inflation” in the sentence in which it appears (institutional, columnist, expert source, etc), and is there any internal variation within newspapers? What would the implications be if different time frames were chosen for the comparison (e.g. monthly or daily)? Why were function/proper nouns removed from the collocate analysis? If the study methodology doesn’t cover such issues, this could be made explicit in the limitations.

More information on other aspects of methods is also necessary, e.g. the authors state they extracted sentences from the corpus, but also talk about normalised frequencies – presumably these frequencies come from full-text data? There were other aspects that also remained unclear to me; thus the description/explanation of the methodology was not sufficiently transparent for readers unfamiliar with the study.

Comments on language/style: The writing is mainly clear, but in the Conclusion there were several sentences that were difficult to understand and that would require rewriting. What exactly was meant remained unclear to this reader. Examples include:

“the structured expression of emotions is paramount when transmitting the importance of economic momentum and the emotions behind the figures.”

“contributes to the discussion of their terminological nature and their communicative and psychological potentiality, as the sentiment conveyed in meaning goes beyond the univocal concept/definition linearity and aseptic nature,”

“relies heavily on a mutating multi-level linguistic edifice”

“The discursive framework emitted by a news item”

It might be good to add [sic] after ‘men’ in this quote to highlight the old-fashioned writing: “If men define situations as real, they are real in their consequences”.

Other than the conclusion, some other stretches that were difficult to parse:

“is an instrument for projecting the sentiment of economic policies in a subliminal way, which we have not yet mastered, but whose potential we are beginning to glimpse”

“rear-view observation of well-acknowledged events (7) to the detriment of an urgent corpus that compiles current affairs offers a more appealing linguistic panorama and provides background knowledge for understanding current historic events.”

The article is also too long in general and could benefit from some general editing, taking out information that is quite general or fairly tangentially related to the article or that could be deleted without changing the main thrust of the argument (e.g. section 2.4 and others). Results could also be reported more concisely (without cutting examples). In addition, the points about metaphor could perhaps be removed, if the focus of this study is on sentiment/emotion rather than metaphor.

**Some comments on some of the underlying assumptions of the study**

There seems to be an indirect or implied assumption that media language informs us directly about (general) language change, shifts in meaning, or that it informs us about speakers’ lexicon (mental representations of words). However, it might be safer to say that this study is limited to analysis of how media language use changes in response to external events, rather than imply that this analysis and its results can inform us about ‘normal’ word usage, changes in word meaning, speakers’ lexicons, and the like. Newspapers are a particular type of institutionalised text with specific language features, which influences the results. For instance, many words will have a neg prosody in newspaper texts but this may not correspond to prosodies in other text types. It would also be good to bring in some corpus linguistic references that have already analysed changes in word meaning in newspaper language and the methods that they have used to do so. This would then allow to relate this study’s methods more specifically to previous research in the area. Previous studies have analysed sentiment shifts over time and have also analysed changing collocates (UFA), but (to my knowledge) no previous studies have attempted to combine the two methods for studying how words are used in the news media over time. If this is indeed the case, it would be useful to state this method innovation explicitly for the reader. More specific information on the methodologies that have been used previously in relevant studies would help, as the current information on this remains fairly vague (and some research is missing, e.g. studies of newspaper language by Antoinette Renouf and colleagues). The above comments would impact on rewriting section 2.1 and 2.2, but also throughout the article (eg points made in the Introduction).

Section 2.3 might work better prior to line 277, where Lingmotif is introduced. The following refs on sentiment analysis could be useful to integrate:

Lei, L., & Liu, D. (2021). *Conducting Sentiment Analysis* (Elements in Corpus Linguistics). Cambridge: Cambridge University Press. doi:10.1017/9781108909679

Taboada, Maite, Sentiment Analysis: An Overview from Linguistics (January 2016). Annual Review of Linguistics, Vol. 2, Issue 1, pp. 325-347, 2016, Available at SSRN: <https://ssrn.com/abstract=2747901> or [http://dx.doi.org/10.1146/annurev-linguistics-011415-040518](https://dx.doi.org/10.1146/annurev-linguistics-011415-040518)

It would be good to bring out some of the shortcomings/critiques of sentiment analysis from a linguistic point of view, and clarify whether any errors in the sentiment analysis were identified through manual checking (as these could influence the results). How accurate was the sentiment analysis on the dataset that was processed here?

**A few other points:**

Amelioration/pejoration is centrally mentioned in the abstract and in early parts of the paper and mentioned again in the Conclusion, but not a single time in the discussion of results. This makes it difficult for the results to be linked clearly as evidence for these phenomena and also raises wrong expectations in readers about what to expect in the discussion of results. The authors could take out these terms or change how the results are written up. The exact same problem occurs with “event word”. Further, this term is never fully explained, unless I missed it, even though it appears to be important to the study. How do the results provide evidence that the term ‘inflation’ has become an event word, as the authors note in the conclusion?

The study assumes that if an item co-occurs with a positive or a negative item, it acquires a positive or negative connotation. However, previous researchers on semantic prosody have argued that this is not always the case, for example in relation to words such as ‘relieve’, ‘soothe’, etc. This would also apply to words such as “ease” that are mentioned in the study.

Refs:

Bednarek, M. (2008) Semantic preference and semantic prosody re-examined. Corpus Linguistics and Linguistic Theory 4/2: 119-139.

Partington, a. (2004) “Utterly content in each other’s company”. Semantic prosody and semantic preference. International Journal of Corpus Linguistics 9, 131–156.

Whitsitt, S. (2005) A critique of the concept of semantic prosody. International Journal of Corpus Linguistics 10, 283–305.

The word ‘speciality language’ that is used repeatedly is not explained and its use was therefore a bit unclear – is the assumption that “inflation” is part of “speciality language”? Or is newspaper language considered speciality language? It would be good to make this clearer to readers.

Minor issues:

Some of the “figures” are tables.

The use of Google Trends is not problematised.

Some claims have no evidence backing, e.g. about intention/perception/effects:

“that translate the experts’ sentiment to influence the public voice.”

“semantic changes in indicators are also very influential factors extralinguistically.”

“that depends on the interests of the editorial board.” (assumes a direct influence/interference of editorial board on linguistic choices made in newspaper texts; also unclear what is meant by ‘editorial board’ with respect to a newspaper)

“and thus be perceived by the readers, who would then agree with the hegemonic discourse.”
