# Supplementary material for: Tracking diachronic sentiment change of economic terms in times of crisis: Connotative fluctuations of ‘inflation’ in the news discourse
Source: PLoS One. 2023 Nov 2;18(11):e0287688. doi: 10.1371/journal.pone.0287688 (PMC10621966; doi:10.1371/journal.pone.0287688)
Supplement: Attachment — Submitted filename: Response to reviewers.docx [file pone.0287688.s003.docx]

The authors would like to thank the reviewers and the Editor in Chief for sharing their thoughtful comments and advice. We consider that the proposed changes and suggestions have greatly contributed to improving our work. The changes and corrections can be found in the updated version of the manuscript highlighted in red colour. The segments that we have removed have been kept in this version (in strikethrough) to facilitate comparison with the older version. In the following paragraphs, we offer a point-by-point response to each of the comments made by the reviewers.

**Reviewer’s comment:** The issue here is that the data (corpus) is not adequately described by the authors. Much more information is necessary to describe the corpus design and composition (of the Great Recession News Corpus) and to evaluate its representativeness. The reader does not know which newspapers are included, how many texts from each newspaper there are per year, the readership and political orientation of the included newspapers, etc. No explanation is given of the file names and what they mean. In lines 303-304 the hypotheses are not spelt out more fully.

**Reply:** This is indeed an important oversight on our part, as we simply referred the reader to the original publication where the corpus is described. We have revised the manuscript to ensure that the corpus is properly described and referenced, as well as to provide more detailed information about previous work in the relevant sections. In the revised version we have reorganised section 3 (“Materials and methods”). It now contains four subsections (3.1 Hypothesis, 3.2 Data sources, 3.3 Instruments, and 3.4 Method). Section 3.2 contains a description of the GRNC with the basic facts needed to understand our research, including the file naming conventions.

**Reviewer’s comment:** Regarding the statistical analysis and methods, while some aspects are fully described, others do rely on readers already being familiar with the relevant stats measure/method (for example, the ‘peaks-and-troughs’ and UFA; Gwet’s AC1 agreement statistic). It would be good if the authors could review what they have written and ensure that all aspects/methods are adequately explained to those who are unfamiliar with them.

**Reply:** Thank you for pointing these issues. We understand that some aspects were not described clearly enough for a wider audience. In the revised version we made sure to provide a detailed account of what was measured and how it was measured, including explanations of the terminology used. Specifically, we provided more information on the peaks-and-troughs technique, the basic principle underlying UFA and Gwet's AC1 methods, as well as the relative frequency values presented in Table 2.

**Reviewer’s comment:** It would also be good if explicit critical method reflection was included, for example in the Conclusion (what can these methods not show us? What are their shortcomings?) […] If the study methodology doesn’t cover such issues, this could be made explicit in the limitations.

**Reply:** Apart from the very much needed clarifications in section 3, we also added a critical reflection on the methods in the last two paragraphs of section 5.

**Reviewer’s comment:** It was also unclear to me whether individual variation (e.g. between newspaper articles, authors, newspapers, newspaper type) was included in the statistical analysis. […] Is there any effect of the type of news item (eg opinion vs news), the voice who produces the word “inflation” in the sentence in which it appears (institutional, columnist, expert source, etc), and is there any internal variation within newspapers?

**Reply:** We have clarified in 3.2 that no individual variation was required at the time of compiling the dataset, as its analysis (e.g., differences between papers or language varieties) would go beyond the scope of this study. It was present in the *future perspectives* subsection of the Conclusions section of the initial draft of our manuscript and we have decided to reintegrate it in the text.

**Reviewer’s comment:** No information on such issues is in the tables/results, which just appears to provide aggregate information per year. What would the implications be if different time frames were chosen for the comparison (e.g. monthly or daily)?

**Reply:** Even if the corpus has by design a monthly sensitivity, in 3.4 we explain that the sentiment peaks-and-troughs were calculated quarterly in Step 2, in order to observe the peaks-and-troughs more clearly (Figs 3 & 4).

In steps 1, 3 and 4, however, we stick to a yearly analysis as it avoids any seasonality in the data. A 36-data point analysis (quarterly) would be too long and tedious for a journal article, especially for UFA.

**Reviewer’s comment:** Why were function/proper nouns removed from the collocate analysis?

**Reply:** Function words and proper nouns were removed from the analysis as they do not inherently convey sentiment and more relevant information would be lost amongst excessive data. We have mentioned this in section 3.2.

**Reviewer’s comment:** More information on other aspects of methods is also necessary, e.g. the authors state they extracted sentences from the corpus, but also talk about normalised frequencies – presumably these frequencies come from full-text data? There were other aspects that also remained unclear to me; thus the description/explanation of the methodology was not sufficiently transparent for readers unfamiliar with the study.

**Reply:** We have addressed this by specifically mentioning that the relative frequencies refer to the number of occurrences per million words in the original corpus (final paragraph of section 3.3).

**Reviewer’s comment:** Comments on language/style: The writing is mainly clear, but in the Conclusion there were several sentences that were difficult to understand and that would require rewriting. What exactly was meant remained unclear to this reader. Examples include:

“the structured expression of emotions is paramount when transmitting the importance of economic momentum and the emotions behind the figures.”

“contributes to the discussion of their terminological nature and their communicative and psychological potentiality, as the sentiment conveyed in meaning goes beyond the univocal concept/definition linearity and aseptic nature,”

“relies heavily on a mutating multi-level linguistic edifice”

“The discursive framework emitted by a news item”

It might be good to add [sic] after ‘men’ in this quote to highlight the old-fashioned writing: “If men define situations as real, they are real in their consequences”.

Other than the conclusion, some other stretches that were difficult to parse:

“is an instrument for projecting the sentiment of economic policies in a subliminal way, which we have not yet mastered, but whose potential we are beginning to glimpse”

“rear-view observation of well-acknowledged events (7) to the detriment of an urgent corpus that compiles current affairs offers a more appealing linguistic panorama and provides background knowledge for understanding current historic events.”

**Reply:** We agree that the style used in these segments may be confusing. We have rephrased these with more readable versions. We believe the original intended message is much clearer now.

**Reviewer’s comment:** The article is also too long in general and could benefit from some general editing, taking out information that is quite general or fairly tangentially related to the article or that could be deleted without changing the main thrust of the argument (e.g. section 2.4 and others). Results could also be reported more concisely (without cutting examples). In addition, the points about metaphor could perhaps be removed, if the focus of this study is on sentiment/emotion rather than metaphor.

**Reply:** We have removed section 2.4, as its most relevant contents are in fact discussed elsewhere in the article. As for the segment on metaphor systems, we agree that, although interesting, it distracts the reader from the main objective of the article, and may cause confusion. Thus, we have also decided to remove it. Also, we have rearranged the examples for a smoother reading.

**Reviewer’s comment: Some comments on some of the underlying assumptions of the study**

There seems to be an indirect or implied assumption that media language informs us directly about (general) language change, shifts in meaning, or that it informs us about speakers’ lexicon (mental representations of words). However, it might be safer to say that this study is limited to analysis of how media language use changes in response to external events, rather than imply that this analysis and its results can inform us about ‘normal’ word usage, changes in word meaning, speakers’ lexicons, and the like.

Newspapers are a particular type of institutionalised text with specific language features, which influences the results. For instance, many words will have a neg prosody in newspaper texts but this may not correspond to prosodies in other text types.

**Reply:** Thank you very much for evidencing this fact, we understand that section 5 lacked a clearer statement on these crucial issues, which were somewhat lost in the conclusions. We have added a paragraph at the beginning of the section where we clearly state these ideas that synthesize the core findings of this research.

**Reviewer’s comment:** It would also be good to bring in some corpus linguistic references that have already analysed changes in word meaning in newspaper language and the methods that they have used to do so. This would then allow to relate this study’s methods more specifically to previous research in the area.

Previous studies have analysed sentiment shifts over time and have also analysed changing collocates (UFA), but (to my knowledge) no previous studies have attempted to combine the two methods for studying how words are used in the news media over time. If this is indeed the case, it would be useful to state this method innovation explicitly for the reader.

More specific information on the methodologies that have been used previously in relevant studies would help, as the current information on this remains fairly vague (and some research is missing, e.g. studies of newspaper language by Antoinette Renouf and colleagues).

The above comments would impact on rewriting section 2.1 and 2.2, but also throughout the article (e.g., points made in the Introduction).

**Reply:** The first paragraph of section 2.2 speaks of the paper by Kutuzov (2018) and the one by Alessi and Partington (2020) which cover extensively the state of the art.

We added more clarifications on the contributions of the paper in the last paragraph of section 1.

**Reviewer’s comment:** Section 2.3 might work better prior to line 277, where Lingmotif is introduced. The following refs on sentiment analysis could be useful to integrate:

Lei, L., & Liu, D. (2021). *Conducting Sentiment Analysis* (Elements in Corpus Linguistics). Cambridge: Cambridge University Press. doi:10.1017/9781108909679

Taboada, Maite, Sentiment Analysis: An Overview from Linguistics (January 2016). Annual Review of Linguistics, Vol. 2, Issue 1, pp. 325-347, 2016, Available at SSRN: <https://ssrn.com/abstract=2747901> or [http://dx.doi.org/10.1146/annurev-linguistics-011415-040518](https://dx.doi.org/10.1146/annurev-linguistics-011415-040518)

It would be good to bring out some of the shortcomings/critiques of sentiment analysis from a linguistic point of view, and clarify whether any errors in the sentiment analysis were identified through manual checking (as these could influence the results). How accurate was the sentiment analysis on the dataset that was processed here?

**Reply:** We have expanded our description of sentiment analysis in an attempt to provide the reader with a wider background (although an in-depth treatment of the topic falls outside the scope of the paper). We have included references to Taboada’s work, which can indeed be used to expand on the linguistic aspects of sentiment analysis. More importantly, we have now included an accuracy performance evaluation of the Lingmotif SA tool on our own data, as we agree that we cannot take for granted results of previous evaluations of the tool with general language (section 3.3).

**A few other points:**

**Reviewer’s comment:** Amelioration/pejoration is centrally mentioned in the abstract and in early parts of the paper and mentioned again in the Conclusion, but not a single time in the discussion of results.

This makes it difficult for the results to be linked clearly as evidence for these phenomena and also raises wrong expectations in readers about what to expect in the discussion of results.

The authors could take out these terms or change how the results are written up. The exact same problem occurs with “event word”. Further, this term is never fully explained, unless I missed it, even though it appears to be important to the study. How do the results provide evidence that the term ‘inflation’ has become an event word, as the authors note in the conclusion?

Reply: We have added more background to Moirand’s concept of “event word” in §1 and linked the evidence of both “event word” and “pejoration/amelioration” to the findings in our data.

**Reviewer’s comment:** The study assumes that if an item co-occurs with a positive or a negative item, it acquires a positive or negative connotation. However, previous researchers on semantic prosody have argued that this is not always the case, for example in relation to words such as ‘relieve’, ‘soothe’, etc. This would also apply to words such as “ease” that are mentioned in the study.

Refs:

Bednarek, M. (2008) Semantic preference and semantic prosody re-examined. Corpus Linguistics and Linguistic Theory 4/2: 119-139.

Partington, a. (2004) “Utterly content in each other’s company”. Semantic prosody and semantic preference. International Journal of Corpus Linguistics 9, 131–156.

Whitsitt, S. (2005) A critique of the concept of semantic prosody. International Journal of Corpus Linguistics 10, 283–305.

**Reply:** Thank you very much. We have expanded section 2.2 and added a paragraph on this topic, as it can help to clarify our statements and added it to our analysis in section 4.

**Reviewer’s comment:** The word ‘speciality language’ that is used repeatedly is not explained and its use was therefore a bit unclear – is the assumption that “inflation” is part of “speciality language”? Or is newspaper language considered speciality language? It would be good to make this clearer to readers.

**Reply:** thanks for pointing this out. We have included an illustration and changed it to the more accepted term “specialized languages”.

Minor issues:

**Reviewer’s comment:** Some of the “figures” are tables.

**Reply:** Thanks, this has been fixed.

**Reviewer’s comment:** The use of Google Trends is not problematised.

**Reply:** Although this issue would indeed benefit from further discussion, we think it would distract the reader from the main narrative.

**Reviewer’s comment:** Some claims have no evidence backing, e.g. about intention/perception/effects:

“that translate the experts’ sentiment to influence the public voice.”

“semantic changes in indicators are also very influential factors extralinguistically.”

“that depends on the interests of the editorial board.” (assumes a direct influence/interference of editorial board on linguistic choices made in newspaper texts; also unclear what is meant by ‘editorial board’ with respect to a newspaper)

“and thus be perceived by the readers, who would then agree with the hegemonic discourse.”

**Reply:** We have restructured the Conclusions to make it less redundant and provided evidence to statements that were not supported by literature.
