# Supplementary material for: Tracking diachronic sentiment change of economic terms in times of crisis: Connotative fluctuations of ‘inflation’ in the news discourse
Source: PLoS One. 2023 Nov 2;18(11):e0287688. doi: 10.1371/journal.pone.0287688 (PMC10621966; doi:10.1371/journal.pone.0287688)
Supplement: Attachment — Submitted filename: Response to reviewers.docx [file pone.0287688.s005.docx]

The authors would like to thank the reviewers and the Editor in Chief for sharing their thoughtful comments and advice. We consider that the proposed changes and suggestions have greatly contributed to improving our work. The changes and corrections can be found in the updated version of the manuscript highlighted in red colour. The segments that we have removed have been kept in this version (in strikethrough) to facilitate comparison with the older version. In the following paragraphs, we offer a point-by-point response to each of the comments made by the reviewers.

**Some final suggestions**

1. **Typos and stylistic issues that could be addressed before finalizing the manuscript:**

Some examples don’t contain any bold to highlight the words of interest. Check and use bold font in all, as appropriate.

**Reviewer:** Not sure about how common/useful some complex phrases are that are used by the authors (e.g. “aseptic description”, “the polyhedral dimensions”, “far from aseptic’); final proof-read might be useful to make language less overly-complex.

**Reply:** Thanks for the suggestion, we simplified these sort of ‘peacock’ terms with more plain language. We also made subtle changes throughout the text to improve reliability.

**Reviewer:** The paragraph in lines 43-48 needs work:

43 explains that newspapers cover special events and use “event words” (*mots événements*)

44 that are often featured on the front page with a headline that indicates the theme of an

45 emotional discourse that typically spans multiple issues and recurs cyclically over time.

- But what are event words exactly? How are they defined? This just tells us how event words are featured, not what they are. In addition, there is no flow/cohesion to the next sentence.

46 Specialized languages (e.g., the language of economy and finance) have their own

47 terminology, expressions, and constructions that are far from univocal and reflect a

48 particular zeitgeist.

**Reply:** We reworked the description of this concept, which is key to understanding our work and was probably not explained in enough detail. We think the current description conveys its intended significance to the reader more accurately.

**Reviewer:** When using “specialised languages” and “specialised texts” etc – just add in brackets what is meant in each instance (e.g. whether you are talking about financial terminology such as *inflation* or specialised texts such as financial news) - just to clarify this for the reader.

**Reply:** Thanks for the suggestion. We have done this.

**Reviewer:** The structural connection between lines 204-212 and what comes before is unclear (cohesion/flow). I would delete this entirely, with the exception of Renouf’s and colleagues work which should be included in the relevant section on how cl methods can be used to study change in newspaper language.

**Reply:** Thanks. We have deleted those lines and moved Renouf’s bit moved it to the introduction section.

**Reviewer:** Lines 694-697 perhaps work better earlier in the introduction rather than in the conclusion as a new point.

**Reply:** We agree. Thanks.

**Reviewer:** Lines 737-740 – I don’t understand the reference to “the discursive framework”. Suggest to delete these two sentences.

**Reply:** Further discussion would require more space, so we think it is a good idea to remove this superfluous information.

**Reviewer:** Typos that I spotted:

274 description of linguistic aspects that intervene in the expression of evaluation)

- See who?

284-285 We consider that terms event words in the news when there is a sudden rise in relative frequency.

- Terms become event words?

415 Figs 4 and 5 plot, respectively, TSS and TSI fluctuations aggregated by the quarterly average.

- Incomplete sentence

Other typos may still be present.

**Reply:** Thanks. We have performed a thorough revision of the text and found and fixed a number of other typos and minor issues. All of them have been highlighted in red.

2. **Content suggestions:**

**Reviewer:** A minor point of clarification on the methodology is needed – adding a sentence regarding step 3 and step 4 – presumably these steps are not just based on the extracted sentences but the full texts? I don’t think this was explicitly mentioned anywhere, whereas the basis for the sentiment analysis (extracted sentences) is now very clear.

**Reply:** You are correct in your assumption. Sorry, we took this info for granted. We clarified this in sections 3.2 and 3.4.

**Reviewer:** There are still various places where the article assumes that change in how words occur in newspapers = semantic change, language change (e.g. lines 64-65; 120-121) even if they clarify this in the Conclusion. Just do another review of the content and adjust the language where necessary (e.g. changing phrase to “semantic change in the news” or “sentiment change in the news”). Section 2.1 still seems to presuppose that what the authors are studying is general semantic change, general language change rather than just studying how the usage of words changed in the time period under investigation. Perhaps the authors could add a clarifying sentence at the end of this section, stating explicitly that they are not studying language change or semantic change in general, but rather in a particular context – newspaper language (as they do in the conclusion). An alternative would be to delete this section entirely, and integrate the explanation of ‘pejoration’ versus ‘amelioration’ elsewhere, where it is relevant, e.g. in the section on semantic prosody. This would also save some space.

**Reply:** We added two introductory sentences in 2.1 that provide clarification. We believe the text now closely describes our intended meaning.

**Reviewer:** Following the review, the authors have just included additional sentence-length summaries of three relevant studies on semantic prosody (Stubbs, Whitsitt, Bednarek), rather than using these (and others) to position themselves explicitly with respect to their own approach.

What underlying assumption is made by the authors? That if *inflation* collocates with negative words, *inflation* acquires a negative connotation and if *inflation* collocates with positive words, *inflation* acquires a positive connotation?

So the assumption would be that in the case of ‘ease inflation’, *inflation* (by itself) has acquired a more positive connotation than in the case of ‘fight inflation’?

So, therefore inflation has now been ‘semantically ameliorated’? I would say that it is this exact claim that is debated by Bednarek, Partington, Whitsitt and others, countering that mere collocation is not always sufficient evidence for connotation and that this also depends on the meaning of the words that collocate (for example, if ‘ease’ has a negative prosody then what is ‘eased’, ‘solved’, ‘fixed’ just as much as what is ‘fought’ etc remains a negative entity). 1

Consider the sentences ‘How to ease inflation’ and ‘How to fight inflation’ – would ‘inflation’ not have a negative connotation in both?

Basically, the point isn’t to include the references just for the sake of having mentioned them, but to engage with the underlying issue regarding how the authors conceptualise/approach this in their own work. In fact, the authors state later (line 515-516) that Whitsitt and Bednarek support their suggestion that ‘ease’ may soften the negative connotation of ‘inflation’, but that is a bit of an imprecise or even inaccurate interpretation of what these authors (and Partington) actually say about words such as ‘ease’.

If the authors want to retain their argument that any collocation with positive words = semantic amelioration and any collocation with negative words = semantic pejoration, this should be explicitly defended against such counter-arguments and reasons given for why the authors make this assumption regardless of the points these three authors have raised. A paragraph (or two) might be sufficient to address this issue in this article.

**Reply:** We have added two paragraphs clarifying our perspective on this major issue and made our position clear on this controversial topic. In a nutshell, from the distant-reading position we assume, we believe that changes in the polarity of the collocates of a term does signal the term’s change in semantic orientation.
