# Supplementary material for: Tracking diachronic sentiment change of economic terms in times of crisis: Connotative fluctuations of ‘inflation’ in the news discourse
Source: PLoS One. 2023 Nov 2;18(11):e0287688. doi: 10.1371/journal.pone.0287688 (PMC10621966; doi:10.1371/journal.pone.0287688)
Supplement: Attachment — Submitted filename: Response to reviewers.docx [file pone.0287688.s006.docx]

The authors would like to thank the reviewers and the Editor in Chief for their prompt response and sharing their comments and advice. We consider that the proposed changes and suggestions have greatly contributed to improving our work.

We ensured that all modifications in the citations have been referenced in the Rebuttal letter.

The changes and corrections can be found in the updated version of the manuscript highlighted in red colour.

We removed the original Fig 5. Former Fig 6 is now Fig 5.

The segments that we have removed have been kept in this version (in strikethrough) to facilitate comparison with the older version. In the following pages, we offer a point-by-point response to each of the comments made by the reviewers.

**Replies to the additional Editor Comments:**

1.
Line 99: "In contrast to the Federal Reserve" -- add "in the USA"
**Reply:** We did it.

2.
Line 109: "that raised the existence" - what does 'raised' mean here? probably a different word is needed

**Reply:** Thanks, we modified the paragraph.

3.
Line 155: "Waldron [11" -- should be "Waldron [18]"
**Reply:** Thank you very much. It seems that Zotero failed to update citations with page numbers and we did not notice this error.

**NOT YET**
4.
Line 161: "We adhere to Lyons’ [12," -- the reference should be "[20,"
**Reply:** Same. Thank you.

5.
Line 167: "[l]anguages.." -- make it "languages..."
**Reply:** Done.

6.
Line 199: "Louw [19, p. 157] defined semantic..." -- Louw reference is [26]
**Reply:** We removed the reference, as it was cited three lines above with no citation in between.

7.
Lines 208-219: except for a short survey of detractors, what is the purpose/message of this paragraph? It is unclear. In the review of R1, a reviewer has also mentioned about this "Basically, the point isn’t to include the references just for the sake of having mentioned them, but to engage with the underlying issue regarding how the authors conceptualise/approach this in their own work."
The current paragraph in R2 is just mentioning detractors of semantic prosody.
The statement on lines 217-219: "The paper emphasizes the need to differentiate between collocational patterning and the connotations associated with a lexical item"
is not clear. Where is this differentiation reflected?
Maybe omit this whole paragraph.

**Reply:** Thanks for the suggestion. We agree that it may cause confusion in reader so we have decided to remove this paragraph.

8.
Line 221-222: "and progressively over time collocates more and more frequently" -- the sentence seems broken around the word 'collocates'. Rewrite this sentence.

**Reply:** We suggest this (225-227):

Finally, we argue that terms can develop semantic prosody as they gain prominence in news discourse following a major event, gaining gradual collocation with new non-domain-specific (i.e., general language) words over time.

9.
Line 240: "topics, and their attributes” [22, p. 259]." -- reference number [22] is not correct. Maybe 36?
**Reply:** We corrected this: [29]

10.
Lines 250-251: "(specifically, Transformers-based models, such as BERT) offer current state-of-the-art results for SA tasks [38]" --
Reference 38 is about machine translation. For sentiment, try a different paper,
maybe <https://aclanthology.org/N19-1035/>
**Reply:** We added the suggested paper to the references. Thanks.

11.
Line 260: "The traditional criticism to machine learning approaches (e.g., [30])" -- wrong reference [30].
**Reply:** We fixed this: [34,35]. Thanks.

12.
Note: Publication [41] (Taboada) is in the bibliographic list, but is not referenced in the manuscript text.
Consider mentioning it in section 2.3

**Reply:** We added this reference ([37]).

13.
Line 303: "is a sudden rise in relative their frequency" --> "their relative frequency"

**Reply:** Thanks, we have corrected this.

14.
Lines 329-330: "We opted for the LogDice statistical measure (14-LogDice(7), L5-R5,C5,NC35),"

a) Please provide a reference to the LogDice original paper
Rychly, P. (2008). A lexicographer-friendly association score. In P. Sojka & A. Horak
(Eds.), Proceedings of recent advances in Slavonic natural language processing,
RASLAN (pp. 6–9). Brno: Masaryk University.

b) Please explain "L5-R5,C5,NC35" - those are not self-evident.

**Reply:** Thank you very much. We added the reference and also clarified this notation in the text.

15.

lines 326-327: "For our research purposes, individual variation (e.g., authors, newspapers) was relevant."
It is not clear how relevant it was, i.e. where is it reflected in the results?
Maybe omit this sentence.
**Reply:** OK, we removed this sentence.

16.
Lines 373-380 (evaluation study):
Manual annotations are into 3 classes (positive, negative, neutral),
but TSS is on the scale 0-100.
Please describe how do you convert TSS values into (positive, negative, neutral)
(using cutoffs ?), so as to get 3-class results that are presented in Table 1.

**Reply:** We modified the following sentence: Three annotators independently labelled the sample dataset as belonging in one of three classes ~~(positive, negative, neutral)~~: positive (TSS>50), negative (TSS<50), neutral (TSS=50).

17.
Lines 383-385: "Sketch Engine [56], a web-based tool that extracts linguistic information, (in our case,
relative frequencies per million tokens, collocations and concordances) from large corpora."
It is not quite clear how you used the Sketch Engine. Did you upload the GRNC to the SketchEngine?
Did you calculate frequencies over whole GRNC, or just the 'inflation' subset, or in other corpora? State it explicitly.

# **Reply:** We added the following sentences (402-405): We uploaded the GRNC to the SketchEngine in XML format so that the subcorpora feature was available. Relative frequency was calculated by dividing the number of occurrences of ‘inflation’ in each yearly subcorpus by the total number of words in the subcorpus, and then multiplying it by 1 million.

18.
Section 3.4 "Method".
This section lists four "steps" of analysis (those are separate analyses),
but it does not provide the results.
The results are listed in section 4.
This arrangement makes it difficult for the reader to follow the logic/description of analyses and their results (due to separation in the manuscript).
Please consider rearranging the text so that each description of analysis is followed by its results,
and then switch to the next set of analyses.

**Reply:** We think that these steps summarize properly our process and anticipate what is to be done. Nevertheless, we added subsections to section 4 to facilitate the following of the methodological process.

19.
Line 413: "analyzed using the peaks-and-troughs [59] technique"
Reference 59 is Gabrielatos&Marchi2012, but that presentation is about keyness, it is not about the peaks-and-troughs method ( peaks-and-troughs are not even mentioned in those slides).
Please cite a proper source.
**Reply:** We added this source to [54]:

Gabrielatos C, McEnery T, Diggle PJ, Baker P. The peaks and troughs of corpus-based contextual analysis. International journal of corpus linguistics. 2012;17: 151–175.

20.
Figures 4 and 5: the figures are presented with clipped range on the Y axes.
Please consider adding a version the Fig.4 chart with the full range of the Y-axis, so readers can get a fuller impression of the fluctuations relative to the full 0-100 range of the scale.

The values on Fig.4 seem to be in the narrow range 40-51, which is very close to the general 'neutral' line of 50.
Or maybe mark on the chart which zones of the Y-axis are negative/neutral/positive
(maybe use the cutoff values from the evaluation study?)

**Reply:** We generated a new plot with a neutral line in TSS=50. We marked positive/negative consecutive points with colors green/red.

We prefer to zoom in on the range of relevant values (TSS 30-70) so that the differences in the fluctuation are more apparent. It is also the default behavior in visualizations of this kind. We attach both plots (Figs A and B), so the difference can be appreciated.

We also added the reference [55] for the regression model:

Wood SN. Generalized Additive Models: An Introduction with R. 2nd ed. Chapman and Hall/CRC; 2017. doi:10.1201/9781315370279

Fig A. TSS GAM, covering a TSS 30-70 range

Fig B. TSS GAM, covering a TSS 0-100 range

21.
Lines 444-463:
"In Fig 4, we identify the first stage in 2007, which corresponds to the stage prior
to the outbreak of the crisis, and a second stage of six years that starts with a pronounced
decline in negative sentiment values in 2008 and remains slightly upward until 2013."

This description does not seem to be corresponding to what I see in Figure 4.
For 2007 the line goes steeply down, which indicates growing negative sentiment.
The growth of negative sentiment slows in 2008. You use the word 'decline'
This is not adequate for two reasons. First, it is incongruent with the graphical depiction - in the chart going-down (or declining) means become more negative,
whereas you mean that negativity trend lessens (so decline in negativity is line-going-up-in-the-chart).
Negativity seems to be stable in 2009 and the change to positive seems to begin in 2010.

**Reply:** We apologize for the confusion caused by the use of the term "decline" in the sentence. You are correct in noting that the term "decline" is inappropriate in this context.

To accurately describe the chart, we can rephrase the sentence as follows

In Fig 4, first we observe a steep increase in negative sentiment in 2007. The second phase (2008-2012) is characterized by a predominant negative sentiment and intermittent stabilization, with a discernible shift to a positive sentiment beginning to take shape around 2010.

For the whole section of lines 444-463, the range of obtained TSS values is very close to 50.
Is 40, and 46-47, really negative or is it still within the lower bounds of 'neutral'?
is 51.21 really positive? or just firmly within neutral zone? The claim that 51.21 is 'positive' seems dubious.
Please state what are the negative/neutral/positive zones for the 0-100 TSS scale.

**Reply:**

In 359-365, we have stated clearly that:

Our study is based on Lingmotif’s main sentiment metric, the Text Sentiment Score (TSS), a quantitative scale in the range of 0-100. It quantifies the sentiment of the analyzed text from extremely negative (0) to extremely positive (100). A value of 50 on the TSS scale is considered neutral. Importantly, Lingmotif arrives at a neutral score in one of two situations: either no sentiment words are found or the positive and negative words found cancel each other out. This implies that even scores slightly higher or lower than 50 are strong indicators of positive or negative polarity.

22.
Figure 6.
Please explain what do the colors red/green/grey mean in figure 6.
The manuscript text seems to disregard the coloring.
What did you try to convey there?

Reply: Sorry, we failed to upload the updated version of the figure (now Fig 5). We clarified this in the caption and in the text.

23.
Lines 485-486: "The term ‘inflation’ in the 2007 sub-corpus occurs in contexts with mainly positive connotations".
How this claim can be reconciled with Fig.4 which shows rapid growth towards more negative sentiment in 2007?
So the overall sentiment was declining (becoming negative) but somehow 'inflation' managed to sit in positive contexts?
This is very strange.
The TSS was computed only over 'inflation' sentences from GRNC, right?

**Reply:** Thanks. We specify in 516-524:

The yearly average score of ‘inflation’ in 2007 (TSS=51.27) is positive. In general, these sentences refer to the daily evolution of economy before the advent of the crisis. Most of the terminal UFA collocates belong in the domain of economics and end in 2007, as more prominent collocates will emerge in the following stage. Even if the averages for the second (TSS=47.18) and third quarter (TSS=40.71) are negative, all of the significant collocates for this stage belong to the domain of economy and none of them convey sentiment. For instance, its most significant collocation is ‘adjust’ (LogDice=10.48), which is used to create equivalences between two different historical prices. An example is shown in example (1) below.

24.
a)
Lines 563-564: "In the third study segment (2013-2015), ‘inflation’ co-occurrs with more positive sentiment words. Specifically, directionality indicators (‘low’, and ‘fall’,..."
Problem here is that neither 'low' nor 'fall' are positive sentiment words.
'Fall' (in the sense of falling) might even have some generic negative connotation.
So how do you derive that statement?

**Reply:** We discussed further the concept of directionality, and modified the description of the 2013-2015 period to improve the flow of legibility.

b)
Lines 567-568: "A cursory glance at the 2013-2015 concordance lines clearly indicates positive results".
Please don't send readers to concordance lines (even if the data is made available). It is also very subjective and vague.
You must support your claims in the body of the manuscript! Use statistics when needed.

**Reply:** We agree. To support these observations, we extracted samples, calculated simple statistics, and added related examples.

25.
Line 588: "In 2015 TSS scores fall to the threshold of negativity."
What is that threshold? Is it strictly "50"? Where is the 'neutral' zone?
**Reply:** As we mentioned in 16, we have solved this issue.

26.
Line 595. Please explain what are "HICP figures".
**Reply:** We clarified what the acronym means in the text.

27.
Almost all the description is section 4 is focused on TSS.
The TSI results are mentioned but mostly ignored.
This creates a startling imbalance.
Please consider saying something about the TSI results.
or maybe remove TSI from this manuscript, consider it for another publication?

**Reply:** We agree that we do not provide a highly detailed account of TSI because of extension issues. We have, thus, considered your suggestion and removed it from the manuscript.
